# 

**Published:** 1887-12

**Authors:** 


### CHESHIRE.

EGREMONT.
T. W. A. Napier

MACCLESFIELD.
T. S. Sheldon

STOCKPORT.
K. Maclean

### CORNWALL.

BODMIN.
T. Mudge

CALSTOCK.
H. T. Woodd

CAMBORNE.
J. Paull

CAMELFORD.
E. Pearce

FOWEY.
E. Carnall

LISKEARD.
J. H. Jenkins

LOSTWITHIEL.
C. Row
W. Row
J. H. Sewart

NEW QUAY.
T. Boyle
W. Urwick

PADSTOW.
H. F. Marley

PENZANCE.
W. S. Bennett

POLRUAN.
W. H. Torbock

PORT ISAAC.
C. Williams.

REDRUTH.
T. B. Carlyon
J. S. Hichens
G. A. Michell

ST. AUSTELL.
W. Mason

ST. JUST.
R. B. Searle

SALTASH.
H. B. Runnalls

TRURO.

J. B. Jackson

E. Rundle

### DEVONSHIRE.

BAMPTON.

T. A. Guinness

BARNSTAPLE.
C. H. Gamble
J. Harper
W. A. G. Laing

BEER ALSTON.
A. R. Steel

BIDEFORD.
W. H. Ackland
J. Thompson

BLACK TORRINGTON.
H. H. Parsloe

BRIXHAM.
G. C. Searle

BROADCLYST.
J. Somer

CHAGFORD.
A. D. Hunt

CHILLINGTON.
F. H. Clarke

CHUDLEIGH.
J. A. Watson

COMBE MARTIN.
G. H. Manning

DAWLISH.
A. de W. Baker
F. M. Cann

DEVONPORT.
G. T. Rolston
J. R. Rolston

EXBRIDGE.
G. F. Sydenham

EXETER.
J. M. Ackland
P. M. Deas
A. W. Kempe
L. Shapter
E. B. Steele-Perkins
L. H. Tosswill

HONITON.
T. W. Shortridge

HORRABRIDGE.

A. P. Prowse

KINGSBRIDGE.
W. H. Webb

LYNTON.
F. C. Berry

MORCHARD BISHOP.

W. J. C. Nourse

NEWTON ABBOT.
E. Haydon
W. G. Scott

OTTERY ST. MARY.
F. A. Gray

PAIGNTON.
J. Alexander

### DEVONSHIRE *(Continued).*

PLYMOUTH.
R. H. Clay
Medical Society

ST. MARY CHURCH.
T. Finch

SALCOMBE.
A. H. Twining

SIDMOUTH.
B. G. Pullin

SOUTH MOLTON.
E. Furse

TAVISTOCK.

M. T. Leamon

TEIGNMOUTH.
R. Gibbs

TIVERTON.
J. S. Smith

TORQUAY.
W. Powell

TOTNES.
D. A. Fraser

UFFCULME.
F. Morgan

WINKLEIGH.
J. H. Norman

### DORSETSHIRE.

BEAMINSTER.
T. P. Daniel

BRIDPORT.
G. M. Evans

CERNE ABBAS.
E. W. Kerr

DORCHESTER.
F. B. Fisher
P. W. MacDonald

LYME REGIS.
J. Reader

PARKSTONE.
J. R. Philpots

POOLE.
J. McNicoll

PORTLAND.
G. H. Lilley

SHERBORNE.
W. H. Williams

STURMINSTER NEWTON.
J. C. Leach

THORNCOMBE.
T. A. Palm

WEYMOUTH.
R. W. Carter

WIMBORNE.
A. J. H. Crespi
C. H. W. Parkinson

### ESSEX.

SOUTHEND.
E. E. Phillips

### GLOUCESTERSHIRE.

BRISTOL.

G. Adams
J. B. Adams
O. Andrews
G. F. Atchley
W. M. Barclay
T. G. L. Baretti
B. J. Baron
J. Beddoe
A. E. Blacker
A. F. Blagg
E. C. Board
J. Broom
G. F. Burder
J. P. Bush
A. Carr
W. F. Carter
J. M. Clarke
E. W. Coathupe
R. W. Coe
T. V. Coker
T. J. Colman
G. G. Corbould
W. Cotton
F. R. Cross
J. Dacre
D. S. Davies
H. R. Dew
N. C. Dobson
C. H. Dowson
W. Dowson
E. L. W. Dunbar
T. F. Edgeworth
C. Elliott
J. F. Elwin
W. R. Etches
J. Ewens
W. J. Fawcett
R. G. Fendick
E. L. Fox
W. J. Fyffe
G. Gardiner
A. N. G. Gibbs
G. A. Gloag
W. C. Goodden
W. G. Grace
L. M. Griffiths
C. D. G. Hailes
A. J. Harrison
W. H. Harsant
C. F. Hawkins
A. G. Hayman
J. C. Heaven
C. K. C. Herapath
H. E. Hetling
F. F. Hills
General Hospital
G. A. Imlay
Royal Infirmary
W. P. Keall
F. P. Lansdown
A. E. A. Lawrence
H. Lawrence
E. L. Lees
Museum and Library
W. E. Lloyd
F. T. B. Logan
W. C. Luffman
H. Marshall
C. E. Matthews
T. O. Mayor
J. S. Metford
J. Milne
C. Newman
W. H. C. Newnham
J. Newstead
T. D. Nicholson
J. A. Norton
T. C. Norton
G. S. Page
G. Parker
J. H. Parry
T. C. Parson
G. C. P. Pauli
W. J. Penny
J. H. Perkins
C. F. Pickering
W. E. Pountney

### GLOUCESTERSHIRE *(Continued).*

BRISTOL *(continued).*

A. Prichard
A. W. Prichard
J. E. Prichard
A. Pring
A. B. Prowse
J. R. Rahilly
G. Rogers
W. B. Roué
C. K. Rudge
H. T. Rudge
H. F. Semple
J. E. Shaw
E. J. Sheppard
W. E. Shorland
E. M. Skerritt
G. M. Smith
J. G. Smith
R. S. Smith
S. Smith
W. H. Spencer
G. M. Stansfeld
C. Steele
F. W. Stoddart
J. Swain
J. G. Swayne
S. H. Swayne
J. Taylor
G. S. Thomson
W. J. Tivy
H. Waldo
C. H. Wallace
E. H. Warner
J. H. Wathen
T. Webster
G. G. D. Willett
P. W. Williams

CHALFORD.
F. C. Palmer

CHELTENHAM.
J. W. Bramwell
J. Bubb
J. E. Gabb
J. C. Gooding
Cheltenham Library
C. J. Newton
L. Winterbotham

CHIPPING SODBURY.
A. Grace

CINDERFORD.
W. C. Heane

CIRENCESTER.
E. C. Cripps
C. P. Hooker

COLEFORD.
L. B. Trotter

DOWNEND.
H. Skelton

DURSLEY.
F. J. Joynes

FAIRFORD.
J. Cornwall

FISHPONDS.
W. Brown
H. R. Mead

FRAMPTON-ON-SEVERN.
T. Watts

GLOUCESTER.

R. W. Batten

E. D. Bower

E. I. Rowe

J. P. Wilton

HAMBROOK.
E. Crossman
W. F. B. Eadon

HAWKESBURY-UPTON.
W. I. Cox

KINGSWOOD.
H. Grace

LYDBROOK.
H. S. Fletcher

LYDNEY.
A. S. Currie

MORETON-IN-MARSH.
L. K. Yelf

SHURDINGTON.
J. Lambe

STAPLE HILL.
W. Murray

STAPLETON.
H. A. Benham
G. Thompson

STOKE BISHOP.
J. Stewart
D. H. Walsh
F. F. Welsh

STONEHOUSE.
G. T. B. Watters

STOW-ON-THE-WOLD.
E. Dening

STROUD.

A. S. Cooke

E. A. Howsin

W. H. Paine

F. W. Storry

TETBURY.
W. Wickham

THORNBURY.
E. M. Grace
W. G. Salmon
T. H. Taylor

WESTBURY-ON-TRYM.
H. Ormerod
A. W. Riddell

WINTERBOURNE.
R. Eager

WOTTON-UNDER-EDGE.
D. H. Forty

### HAMPSHIRE.

BOURNEMOUTH.
D. C. Embleton
E. P. Philpots

LYMINGTON.
J. Rendall

SOUTHSEA.
H. Rundle

### HEREFORDSHIRE.

HEREFORD.
W. C. Whitfeld

ROSS.
J. W. Norman

### HERTFORDSHIRE.

CHESHUNT.
W. B. Kesteven

ST. ALBANS.
A. H. Boys

### LANCASHIRE.

LIVERPOOL.
Medical Institution

POULTON-LE-FYLDE.
P. H. Day

### MIDDLESEX.

LONDON.

L. S. Beale
L. Browne
H. Cooper
E. East
J. F. Evans
J. M. Fothergill
A. P. Gould
G. S. Gregory
M. Grigor
G. W. Isaac
C. M. Jessop
J. Lister
M. W. H. Russell
W. J. Seward
Royal Med. and Chir. Society
G. D. Thomas
H. C. Thurston
J. E. Trask

SHEPPERTON.
J. G. Davey

TOTTENHAM.
W. H. Plaister

### MONMOUTHSHIRE.

ABERGAVENNY.
S. H. Steel

CAERLEON.
J. A. Morris

EBBW VALE.
J. W. Davies

NEWPORT.
W. Basset
H. R. Hudson
O. E. B. Marsh
A. G. Thomas
H. E. Williams

PONTYPOOL.
S. B. Mason
E. S. Wood

RHYMNEY.
T. H. Redwood

RISCA.
G. B. Robathan

TREDEGAR.
G. A. Brown

### NORTHAMPTONSHIRE.

KETTERING.
R. E. Burges

### NOTTINGHAMSHIRE.

MANSFIELD.
H. H. Tomkins

### OXFORDSHIRE.

OXFORD.
R. W. Doyne

### SOMERSETSHIRE.

BANWELL.
A. J. Bisdée

BATH.

W. B. Beatson
E. J. Cave
T. Cole
F. S. Cowan
R. Davis
A. E. W. Fox
H. W. Freeman
H. F. A. Goodridge
T. B. Goss
F. K. Green
F. P. Hoblyn
H. C. Hopkins
L. J. King
T. P. Lowe
F. Mason
G. Norman
R. J. H. Scott
E. Skeate
J. K. Spender
L. Weatherly
C. Wilson

BECKINGTON.
W. G. Evans

BRIDGWATER.
F. J. C. Parsons
J. B. Sincock

BRISLINGTON.
B. B. Fox
C. H. Fox

BRUTON.
F. Stockwell

BURNHAM.
A. W. C. Peskett

CHEDDAR.
R. W. Statham

CHEW MAGNA.
E. T. Hale

CLEVEDON.
T. Davis
G. F. P. Pizey
S. Skinner

COMPTON MARTIN.
W. F. Lovell

DULVERTON.
R. J. Collyns

### SOMERSETSHIRE *(Continued).*

FROME.
E. F. H. Burroughs
F. Parsons
F. E. Pearse

GLASTONBURY.
J. A. Bright

ILMINSTER.
C. Munden

KEYNSHAM.
N. Crisp
J. Lodge
R. W. Thomas

LANGPORT.
J. Morgan

LONG ASHTON.
J. Fuller

MELLS.
G. Terry

MIDSOMER NORTON.
E. Blacker
A. Waugh

MILBORNE PORT.
J. F. Royle

MILVERTON.
C. Randolph

MINEHEAD.
T. Clark
T. J. Ollerhead

NORTH PETHERTON.
W. J. Todd

PORTISHEAD.
J. Lidderdale

RIDGWAY.
W. Duncan

SHEPTON MALLET.
B. N. Hyatt

SOUTH PETHERTON.
W. H. Walter

STREET.
A. Clarke

TAUNTON.
H. J. Alford
J. Carey
W. Liddon

TEMPLE CLOUD.
T. Martin

TWERTON-ON-AVON.
J. Wigmore

WEDMORE.
J. Ford
R. P. Tyley

WELLINGTON.
J. Meredith
T. E. P. Prideaux

WELLS.

W. Fairbanks

A. L. Wade

WESTON-SUPER-MARE.
G. E. Alford
G. B. Fraser
E. F. Martin
W. H. G. Phelps
G. F. Rossiter
R. Roxburgh
F. W. S. Wicksteed

WRINGTON.
H. W. Collins

YATTON.
A. de C. Lyons

YEOVIL.
P. S. H. Colmer
C. J. Marsh
E. Vernon

### STAFFORDSHIRE.

STAFFORD.
J. Scott

### SURREY.

CHERTSEY.

W. A. Moynan

### WARWICKSHIRE.

BIRMINGHAM.
L. Tait

### WILTSHIRE.

BRADFORD-ON-AVON.
W. D. Lovell

BURBAGE.
A. E. N. Browne

CALNE.
W. S. Batten

CHIPPENHAM.
G. H. Daly

DEVIZES.
T. B. Anstie
C. Eddowes

DOWNTON.
G. W. Whiteley

FOVANT.
C. Clay

LACOCK.
J. H. Crisp

MALMESBURY.
R. Kinneir

SALISBURY.
H. Coates
B. C. Gowing
L. S. Luckham
H. J. Manning

STRATTON ST. MARGARET.
J. Fernie

SWINDON.
J. B. Fry
W. Howse
J. C. Maclean

TROWBRIDGE.
N. V. Wise

WARMINSTER.
J. Hinton
R. L. Willcox

WILTON.
W. Pippette

WOOTTON BASSETT.
J. M. Kirkman

### YORKSHIRE.

DONCASTER.
F. Penny

### Ireland.

### ANTRIM.

BELFAST.

J. W. Browne

### Scotland.

### ABERDEENSHIRE.

ABERDEEN.

A. Ogston

### EDINBURGHSHIRE.

EDINBURGH.

Y. J. Pentland

### Wales.

### BRECONSHIRE.

BRECON.

A. White

BRYNMAWR.

G. H. Browne

TALGARTH.

W. Howells

SENNY BRIDGE.

W. R. Jones

### CARDIGANSHIRE.

ABERYSTWITH.

R. Gilbertson

LAMPETER.

E. C. Davies

NEW QUAY.

E. R. Jones

### CARMARTHENSHIRE.

CARMARTHEN.

G. J. Hearder

FERRYSIDE.

P. Williams

PENCADER.

E. Harries

LLANELLY.

D. J. Williams

### GLAMORGANSHIRE.

ABERAMAN.

J. B. James

ABERDARE.

E. Jones

CAERPHILLY.

J. Lewellyn

CARDIFF.

G. W. Grote
J. Milward
W. Morgan
W. Price
A. Sheen
Medical Society
C. T. Vachell
H. R. Vachell

COWBRIDGE.

J. W. Phillips

MERTHYR TYDVIL.

J. L. W. Ward

PENARTH.

A. Devonald

REYNOLDSTONE.

H. V. Ellis

SWANSEA.

L. Barnett
G. Padley
J. A. Rawlings
J. S. H. Roberts
J. Thomas

### MONTGOMERYSHIRE.

LLANSAINTFFRAID.

T. Edwardes

### PEMBROKESHIRE.

TENBY.

B. Kendall

### INDEX.

Acetanilde *(Extract)*, 54.
Actinomycosis *(Extract)*, 45.
Albuminuria in syphilitic patients *(Extract)*, 45.
Alopecia areata *(Extract)*, 218.
Aneurism of aortic arch, Case of—Dr. H. F. A. Goodridge, 30; of orbit—A. W. Prichard, 267.
Animal alkaloids — Sir W. Aitken *(Review)*, 198; Dr. Brown *(Review)*, 198.
Animal biology—C. L. Morgan *(Review)*, 273.
Annual, The medical *(Review)*, 119.
Antipyrin in rheumatism *(Extract)*, 48.
Anus, Treatment of fissure of *(Extracts)*, 150, 291.
Aphasia, Sensory *(Extract)*, 220.

Bacteria, Photography of—Dr. E. M. Crookshank *(Review)*, 279.
Bacteriology, Manual of—Dr. E. M. Crookshank *(Review)*, 279.
Ball, C. B.—Diseases of rectum and anus *(Review)*, 276.
Bastian, Dr. H. C.—Paralyses *(Review)*, 64.
Benzoin in treatment of ulcers *(Extract)*, 218.
Brain, Diseases of—Dr. C. W. Suckling *(Review)*, 277.
Browne, L.—The throat and its diseases *(Review)*, 197.
Butlin, H. T.—The operative surgery of malignant disease *(Review)*, 278.

Carbolic acid, Goitre treated by *(Extract)*, 46.
Children, Treatment of disease in—Dr. A Money *(Review)*, 280.
Chloral, Therapeutical applications of *(Extract)*, 149.
Cholera, Treatment of *(Extract)*, 54.
Cirrhosis, Treatment of *(Extract)*, 288.
Clarke, Dr. J. M.—Graves's disease, 17; peripheral neuritis, 73.
Club-foot, Congenital—R. W. Parker *(Review)*, 118.
Cocaine in lithotrity *(Extract)*, 137; in whooping-cough *(Extract)*, 149.
Consumption, Climatic treatment of —Dr. J. A. Lindsay *(Review)*, 272.
Cooper, A.—Diseases of rectum *(Review)*, 272.
Copper, Biological action of *(Extract)*, 211.
Craniotomy to be abolished? Ought —Dr. J. G. Swayne, 1.
Crookshank, Dr. E. M.—Manual of bacteriology; Photography of bacteria *(Reviews)*, 279.
Cross, F. R.—Case of tumour in orbit, 189.
Croup, Statistics of *(Extract)*, 220.

Diabetes, Morphine in (*Extract*), 47.
Diet in its relation to therapeutics (*Extract*), 50.
Diphtheria, Statistics of (*Extract*), 220; treatment of (*Extract*), 51.
Doran, A.—Gynæcological operations (*Review*), 275.
Drumine (*Extract*), 49.

Ear, Case of suppurating middle—W. H. Harsant, 37.
Ear, Diseases of—Dr. A. Hartmann (*Review*), 67; H. M. Jones (*Review*), 276.
Electrical and anatomical demonstrations—Dr. H. Tibbits (*Review*), 194.
Endoscopy (*Extract*), 289.
Enteric fever, Treatment of (*Extract*), 53.

Ergot for headaches (*Extract*), 46.
Erythrasma (*Extract*), 55.
Ether-injections, Subcutaneous (*Extract*), 221.
Eye, Histology of—Dr. C. F. Pollock (*Review*), 69; Refraction of—Dr. G. F. Helm (*Review*), 195.

Friedrich's disease (*Extract*), 215.

Glycosuria in surgical practice (*Extract*), 132.
Goitre, A case of cystic—Dr. C. T. Vachell, 264.
Goitre, Endemic (*Extract*), 212.
Goitre treated by carbolic acid (*Extract*), 46.
Gonorrhœal orchitis, Treatment of (*Extract*), 292.
Goodridge, Dr. H. F. A.—Case of aneurism of aortic arch, 30.
Gordon, Dr. C. A.—Inoculation for rabies and hydrophobia (*Review*), 196.
Graves's disease—Dr. J. M. Clarke, 17.
Griffiths, L. M.—Shakspere and the medical sciences, 225.
Gynæcological operations.—A. Doran (*Review*), 275.

Hæmorrhoids, Treatment of (*Extract*), 291.
Harrison, R.—Surgical disorders of urinary organs (*Review*), 145.
Harsant, W. H.—Case of suppurating middle ear, 37.
Hartmann, Dr. A.—Diseases of ear (*Review*), 67.
Headaches, Ergot for (*Extract*), 46.
Heart, Mobility of the (*Extract*), 56.
Heart, Treatment of valvular disease of—Dr. A. E. Sansom (*Review*), 65.
Helm, Dr. G. F.—Refraction of eye (*Review*), 195.
Hermaphroditism, Case of (*Extract*), 221.
Hernia, Strangulated (*Extract*), 134.
Hernia by subcutaneous injection, Treatment of (*Extract*), 216.
Hoarseness—Dr. B. J. Baron, 163.
Hospital, A travelling (*Extract*), 56.
Hutchinson, J.—Syphilis (*Review*), 274.
Hydatid cyst, Unusual site of (*Extract*), 216.
Hydrarthrus (*Extract*), 132.
Hydrotherapeutics, Journal of (*Review*), 120.
Hypnotics, Some new—Dr. P. MacDonald, 257.

Inoculation for rabies and hydrophobia—Dr. C. A. Gordon (*Review*), 196.

Jessop, C. M.—Latent rheumatism, 153.
Joints, Diseases of—H. Marsh (*Review*), 121.
Jones, Dr. H. M.—Diseases of ear (*Review*), 276.

Keetley, C. B.—Index of surgery (*Review*), 277.
Koprostatic reflex neuroses (*Extract*), 216.

Lanolin salves (*Extract*), 49.
Laryngeal œdema (*Extract*), 56.
Laryngeal phthisis, Treatment of (*Extract*), 143.
Laryngology, Journal of (*Review*), 69.
Laryngoscope in diagnosis (*Extract*), 139.
Larynx, Cancer of (*Extract*), 292; excision of (*Extract*), 294; intubation of (*Extracts*), 145, 146; tuberculous growths in (*Extract*), 144.
Lindsay, Dr. J. A.—Climatic treatment of consumption (*Review*), 272.
Lithotrity, Cocaine in (*Extract*), 137.

MacDonald, Dr. P. W.—Some new hypnotics, 257.
Malignant disease, Operative surgery of—H. T. Butlin (*Review*), 278.
Marsh, H.—Diseases of joints (*Review*), 121.
Medicine, Text-book of—Dr. A. Strümpell (*Review*), 122.
Money, Dr. A.—Treatment of disease in children (*Review*), 280.
Morbus coxæ, Rectal examination in (*Extract*), 133.
Morgan, C. L.—Animal biology (*Review*), 273.
Morphine in diabetes (*Extract*), 47.
Morton, Dr. J.—Treatment of spina bifida (*Review*), 123.

Moullin, C. W. M.—Sprains (*Review*), 273.
Muscular atrophy, Case of—C. Williams, 103.

Nasal diphtheria, Treatment of (*Extract*), 141.
Nasal polypus — Dr. E. Woakes (*Review*), 281.
Nasal septum, Deformities of (*Extract*), 141.
Neuralgias, Treatment of (*Extract*), 148.
Neuritis, Peripheral—Dr. J. M. Clarke, 73.
Nitro-glycerine in mitral stenosis (*Extract*), 217.

Ophthalmitis, Sympathetic (*Extract*), 219.
Orbit, Case of tumour in—F. R. Cross, 189.

Pancreas, The surgery of the (*Extract*), 135.
Paralyses—Dr. H. C. Bastian (*Review*), 64.
Paralysis (*Extract*), 136.
Parker, R. W.—Congenital club-foot (*Review*), 118.
Pathology, Practical — Dr. J. L. Steven (*Review*), 117.
Penny, W. J.—Rhinitis, 95.
Pericardium, Adherent (*Extract*), 214.
Pleural cavity, Washing out the (*Extract*), 213.
Pollock, Dr. C. F.—Histology of eye (*Review*), 69.
Preparations for the sick, Notes on —58, 124, 201, 282.
Prichard, A. W.—A case of aneurism of orbit, 267.
Pullin, B. G.—Treatment of warts, 269.

Quillaja saponaria (*Extract*), 50.

Raynaud's disease (*Extract*), 43.
Rectal injections in cystitis (*Extract*), 217.
Rectum and anus, Diseases of—C. B. Ball (*Review*), 276.
Rectum, Diseases of—A. Cooper (*Review*), 272.
Retinal hæmorrhage in anæmia (*Extract*), 219.
Rheumatism, Antipyrin in (*Extract*), 48.
Rheumatism, Latent—C. M. Jessop, 153.
Rhinitis—W. J. Penny, 95.
Robinson, Dr. T.—Diagnosis and treatment of syphilis (*Review*), 117.
Round ligaments, Shortening the (*Extract*), 139.

Sansom, Dr. A. E.—Treatment of valvular disease of heart (*Review*), 65.
Scarlatina, Three attacks of (*Extract*), 44; surgical, 290.
Scraping in surgery (*Extract*), 150.
Scraps, 71, 151, 223, 295.
Shakspere and the medical sciences —L. M. Griffiths, 225.
Shore, Dr. T. W.—Vegetable biology (*Review*), 194.
Skin irritation, Dyed hosiery and (*Extract*), 55.
Smith, J. G.—Cases of stone in the bladder, 177.
Sodium chloride (*Extract*), 43; in Bright's disease (*Extract*), 49.
Spina bifida, Treatment of—Dr. J. Morton (*Review*), 123.
Sprains—C. W. M. Moullin (*Review*), 273.
Steven, Dr. J. L.—Practical pathology (*Review*), 117.
Stomach, The (*Extract*), 211; treatment of dilatation of (*Extract*), 146.
Stone in the bladder, Cases of—J. G. Smith, 177.
Strümpell, Dr. A.—Text-book of medicine (*Review*), 122.
Suckling, Dr. C. W.—Diseases of brain (*Review*), 277.
Surgery, Index of—C. B. Keetley (*Review*), 277.
Swayne, Dr. J. G.—Ought craniotomy to be abolished? 1.
Syphilis—J. Hutchinson (*Review*), 274.
Syphilis, Diagnosis and treatment of—Dr. T. Robinson (*Review*), 117.
Syphilis of tonsils, Primary (*Extract*), 140.
Syphilitic patients, Albuminuria in (*Extract*), 45.

Tæniafuge, Thymol as a (*Extract*), 46.
Therapeutics, Diet in its relation to (*Extract*), 50.
Throat and its diseases, The—L. Browne (*Review*), 197.
Thymol as a tæniafuge (*Extract*), 46.
Tibbits, Dr. H.—Electrical and anatomical demonstrations (*Review*), 194.
Trichlorophenol (*Extract*), 49.
Tuberculosis of the bulb in a child (*Extract*), 287.

Ulcers, Benzoin in treatment of (*Extract*), 218.
Urinary organs, Surgical disorders of—R. Harrison (*Review*), 195.

Vachell, Dr. C. T.—A case of cystic goitre, 264.
Vegetable biology.—Dr. F. W. Shore (*Review*), 194.

Warts, Treatment of—B. G. Pullin, 269.
Whooping-cough, Cocaine in (*Extract*), 149.
Williams, C.—Case of muscular atrophy, 103.
Woakes, Dr. E.—Nasal polypus (*Review*), 281.

Year-book of scientific societies (*Review*), 120; of treatment (*Review*), 119.

J. W. Arrowsmith, Printer, Quay Street, Bristol.

